# 3D-Printed Triply Periodic Minimal Surface Ceramic Scaffold Loaded With Bone Morphogenetic Protein-2 and Zoledronic for Cranium Defect Repairment

**DOI:** 10.1155/term/9964384

**Published:** 2025-05-26

**Authors:** Junteng Yan, Shuhao Qi, Yiwei Zhao, Peng Tian, Ning Kong, Weigang Ma, Peng Yan, Jiewen Zhang, Xu Gao, Huanshuai Guan, Pei Yang, Qin Lian, Kunzheng Wang

**Affiliations:** ^1^Department of Bone and Joint Surgery, Second Affiliated Hospital of Xi'an Jiaotong University, Xi'an 710054, Shaanxi, China; ^2^State Key Laboratory for Manufacturing System Engineering, Xi'an Jiaotong University, Xi'an 710049, China

**Keywords:** additive manufacturing, bioactive materials, digital light processing, osteogenesis, rabbit cranial defects, tissue engineering, triply periodic minimal surface

## Abstract

Managing large, critical-sized bone defects poses a complex challenge, especially when autografts are impractical due to their size and limited availability. In such situations, the development of synthetic bone implants becomes crucial. These implants can be carefully designed and manufactured as potential bone substitutes, offering controlled parameters such as porosity, hardness, and osteogenic cues. In this study, we employed digital light processing (DLP) technology to construct an alumina ceramic scaffold featuring a triply periodic minimal surface (TPMS) structure for bone transplantation. The scaffold was filled with type I collagen to enhance cell infiltration [1], thereby increasing the total surface area. In addition, type I collagen is a carrier for both bone morphogenetic protein-2 (BMP-2) and zoledronic acid (ZA). Using a clinically relevant rabbit cranium defect model, the scaffold underwent in vivo assessment for its functionality in repairing critical-sized bone defect (approximately 8 mm). Four groups of animal experiments were carried out including the control group, the gyroid scaffold group, the type I collagen-loaded scaffold group, and the bioactive factor-functionalized scaffold group. Our animal-based study results revealed that the gyroid scaffold, functionalized with bioactive molecules, provided a conductive surface for promoting increased bone formation and enhancing the healing process in critical-sized long bone and cranium defects. These findings offer preclinical evidence, supporting the use of a TPMS structure composite scaffold and present compelling support for its application as an advanced synthetic bone substitute in the future.

## 1. Introduction

Craniofacial defects can arise from diverse factors, such as craniofacial reconstruction surgery, tumor removal, congenital diseases, or accidental traumatic incidents [2]. Bones, endowed with inherent regenerative capabilities, can naturally restore the structure and cellular composition of the affected area through a series of coordinated activities involving resorption or remodeling. The bone healing process engages various intricate signaling pathways, including Wnts, bone morphogenic proteins (BMPs), and fibroblast growth factors (FGFs), which play a vital role in embryonic bone development [3, 4]. The choice between autografts, support systems, or bone replacements is influenced by the extent and size of the defect resulting from a traumatic event. During the bone defect healing process, when the size of the defect is smaller than 2–2.5 times the diameter of the affected bone, spontaneous healing often occurs without the need for significant intervention. However, defects surpassing this size cannot heal spontaneously and are categorized as critical defects [5]. For instance, in humans, a tibial defect of 2.5 cm is considered critical [6], while the threshold for femur defects is much higher (> 6 cm) [7]. In craniofacial surgery, a defect of 25 cm^2^ is regarded as challenging [8].

Currently, the primary approaches to address significant bone defects are autologous bone grafting [9], allogeneic bone grafting [10], and bone tissue engineering [11]. Autografts are constrained by their origin, and allografts encounter immune rejection [12], limiting their broader use. Nevertheless, bone tissue engineering has emerged as an effective method for bone repair [13]. While numerous tissue engineering scaffolds have been created, most have basic structures [14] and cannot facilitate the spatial organization of multiple cells. A specific three-dimensional (3D) structure is crucial for simulating tissues and regulating cellular processes, including cell distribution and multicellular interactions [15]. Hence, the production of bone tissue engineering scaffolds that incorporate both multiple cell delivery and intricate morphology resembling natural bone tissue remains a challenging aspect of bone tissue regeneration [16]. The geometric shape of the tissue engineering scaffold plays a vital role in influencing the biological response mechanism [17]. In this context, concave areas are deemed more conducive to tissue formation when compared to convex areas [18, 19]. A reduced curvature of the concave surface facilitates cell invasion by promoting stress on the cell skeleton, thereby encouraging cell migration and tissue growth [19]. Triply periodic minimal surfaces (TPMS) refer to minimal surfaces with crystal structures that repetitively extend in 3D space [20]. A minimal surface is characterized by an average curvature of zero. Common TPMS encompass structures such as gyroids, diamond, and Schwartz-P surfaces. Gyroid structures, in particular, exhibit ample concave surfaces, structural uniformity, and robust interconnectivity. Additionally, these structures resemble trabecular bone and offer controllable porosity [21]. Relative studies have shown that compared with traditional lattice structures, the extraordinary interconnectivity and surface-to-volume ratio of TPMS structures [22] make them ideal structures for conducting new bone ingrowth [23] and promoting cell adhesion, proliferation, and differentiation in tissue engineering [24].

Despite utilizing various methods like freeze casting [25] and layer-by-layer assembly [26] for producing biomimetic biomaterials in tissue engineering, traditional manufacturing techniques encounter challenges in generating intricate structures. In this context, 3D printing emerges as a promising avenue for fabricating materials with customized structures [27], reflecting those found in natural tissues. This technology introduces an innovative method for dynamically adjusting the parameters of the designed tissue engineering scaffold, including specific surface area, wall thickness, and porosity [28]. Presently, a variety of 3D printing technologies, including laser powder bed fusion [29], fused deposition modeling [30], direct ink writing (DIW) [31], and stereolithography [32], have been employed in manufacturing bone tissue engineering scaffolds. Nonetheless, existing 3D printing technologies face limitations such as material selection, resolution, and printing speed [33]. Specifically, when dealing with TPMS structures, the discontinuous nature of these structures on the cross section poses challenges in organizing the printing path, particularly in techniques like DIW [31]. The laser powder bed fusion [29] method often fails to meet the precise printing accuracy required for complex gyroid structures, leading to challenges in replicating fine details on curved surfaces [29]. In contrast, digital light processing (DLP) technology employs the projection of sectional images, eliminating the need for the establishment of a specific printing path. By employing DLP technology, the intricate gyroid structure can be successfully printed with high dimensional accuracy [23]. Consequently, biomimetic ceramic scaffolds can be efficiently produced. This approach offers a rapid, high-precision, and reliable strategy for creating structurally diverse bio-ceramic scaffolds from a single precursor slurry through a one-step process. The programmable TPMS structural designs allow customization to control porosity and specific surface area by adjusting cell thickness and the number of cells.

While there have been studies on the promotion of bone regeneration using TPMS scaffolds, many of these studies have been limited to in vitro assessment, with a few involving animal studies to validate the in vivo applicability of such scaffolds. Additionally, there is a lack of research on the interaction between the TPMS structure and bone-inducing factors, such as bone morphogenetic proteins (BMPs) or zoledronic acid (ZA). Some studies have shown that the combined application of BMP-2 and ZA can better inhibit the bone resorption of osteoclasts, while BMP-2 can enhance the osteogenic effect of osteoblasts and increase the growth rate of bone trabeculae [34, 35]. Specifically, understanding how a gyroid scaffold loaded with osteoinductive factors promotes tissue growth remains an unexplored area in the current literature. Other studies have indicated that incorporating BMP and ZA into hydrogel and gelatin scaffolds enhances bone formation [36]. However, scaffolds utilizing these materials lack adequate mechanical strength, particularly for addressing larger bone defects. Drawing inspiration from the trabecular bone's structure and function, this study employed DLP-based 3D printing technology to fabricate gyroid structural models. These models were designed considering the impact of porosity and scaffold surface area on bone regeneration.

Consequently, a simulated gyroid bio-ceramic scaffold was successfully prepared. To facilitate the 120 days of animal experiment, this study employed TM-DAR alumina material as the scaffold due to its robust chemical stability. Through the utilization of DLP to fabricate gyroid and the subsequent functionalization of these scaffolds with BMP and ZA, the study aimed to achieve scaffolds with superior mechanical properties and enhanced conditions for bone formation compared to those composed of single scaffold types. Furthermore, the bone growth surface was increased by incorporating naturally extracted rat tail collagen. The in vivo performance of the resulting composite-functionalized scaffold was assessed using a critical size bone defect model of the cranium of the Chinese white rabbit. The study also examined the influence of the gyroid scaffold structure on the growth pattern of bone tissue.

This study confirms that the gyroid model effectively enhances bone regeneration in TPMS structures, with the guiding surface and microporous structure significantly influencing cell infiltration, remodeling, and bone healing activities. Moreover, the bone conduction surface area can be augmented through the infiltration of type I collagen, and the scaffold can be endowed with drug-loading capability. Building upon this, the functionalization of the scaffolds with bone-inducing factors (BMP and ZA) further intensified the bone regeneration performance of the scaffolds.

## 2. Materials and Methods

### 2.1. Printing Materials and Preparation Techniques

**Figure 1:**
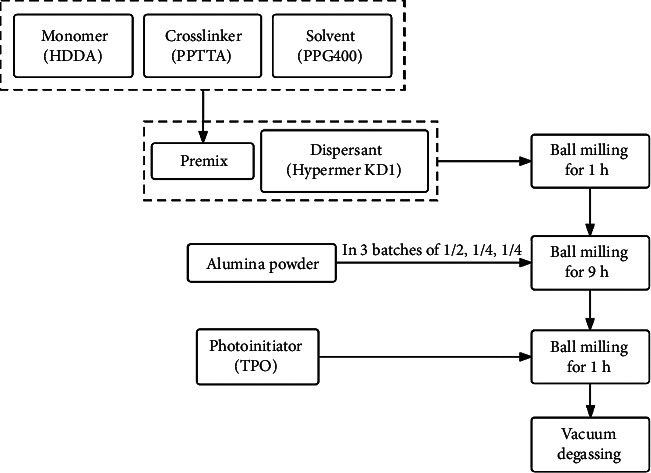
Preparation process of ceramic slurry.

The alumina powder utilized in this study (*d*_50_ = 150 nm, product number: TM-DAR) was supplied by TAIMEI Chemicals (Tokyo, Japan). Regarding the photosensitive polymer, low-viscosity 1,6-hexanediol diacrylate (HDDA) with two functional groups is chosen as the monomer for polymerization, and pentaerythritol tetra-acrylate (PPTTA) with four functional groups is selected as the crosslinker to promote polymerization. These materials were obtained from DSM-AGI, Taiwan. Polypropylene glycol (PPG) with a molecular weight of 400 was purchased from Aladdin, Shanghai, China, and was introduced into the monomeric matrix as a nonreactive solvent to regulate the viscosity of the slurry. To attain high solid loading photocurable suspensions and enhance the dispersion of ceramic powder within the liquid resin mixture, a polyester polymer, Hypermer KD1, obtained from Croda Trading, Shanghai, China, served as a dispersant. DAROCUR-TPO (diphenyl (2,4,6-trimethylbenzoyl)-phosphine oxide, Macklin, Shanghai, China) was employed as a photoinitiator. A photocurable Al_2_O_3_ suspension with a ceramic content of 40 vol.% was prepared through the following steps (as shown in Figure 1). Initially, the monomer (HDDA) and crosslinking agent (PPTTA) were combined with the solvent (PPG) to obtain a premix. Subsequently, the dispersant (Hypermer KD1) was added, and ball milling was conducted. The Al_2_O_3_ ceramic powder was added in three batches of 1/2, 1/4, and 1/4 of the total weight. After 9 h of ball milling, photoinitiator TPOs were introduced and another ball milling for 1 h was conducted. Afterward, the ceramic material underwent a vacuum degassing process to eliminate microbubbles in the slurry. The composition of the alumina ceramic slurry is listed in Table 1. The solid volume fraction of the prepared ceramic slurry was 40%, and its viscosity was measured 3.68 Pa·s at a shear rate of 50 s^−1^ at 25°C, rendering it suitable for the printing process.

### 2.2. Structural Design of Scaffold

The gyroid structure, belonging to the category of TPMS, can be generated through a mathematical function as indicated below:

(1) $$\begin{array}{c} \cos\left(x\right)\times \sin\left(y\right)+\cos\left(y\right)\times \sin\left(z\right)+\cos\left(z\right)\times \sin\left(x\right)=C. \end{array}$$

Equation (1) describes the structure of the minimum cell unit of the gyroid feature. In this equation, *C* denotes a constant that depends on the size of the structure. This minimum cell unit was replicated along the *X*, *Y*, and *Z* coordinates to achieve the comprehensive 3D structure. A Python program generated 3D structures using equation (1), incorporating a specified wall thickness. To fulfil implantation criteria, scaffolds with a columnar TPMS structure were precisely designed, featuring a diameter of 8 mm and a height of 2.5 mm. To ensure the functionality of the TPMS structure in the scaffold, the number of unit cells in the structure was kept at a minimum of 3. The scaffolds were designed with a targeted porosity of 60%, and the wall thickness of the structure was determined based on this specified porosity.

### 2.3. Procedure for 3D Printing and Postprocessing

**Figure 2:**
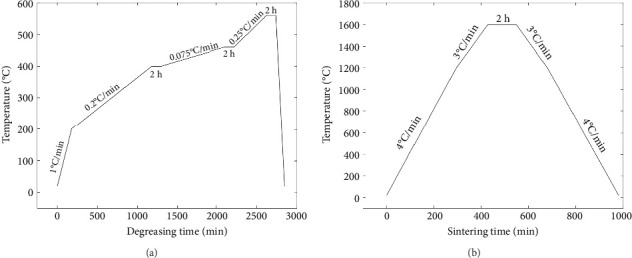
Temperature curve of degreasing and sintering process. (a) Degreasing process. (b) Sintering process.

The functional scaffold was printed using a bottom-up DLP printer (Craftsman 150 3D Printer, Shaanxi Ketao-AM Technology Co., Ltd., China). The printer's projector employs a light source with a wavelength of 405 nm to cure the ceramic slurry. To account for the shrinkage during the degreasing and sintering process, it was necessary to measure the shrinkage ratio of the developed ceramic material beforehand. Initially, a cubic part was printed to assess shrinkage, revealing 23.8% in the *X*–*Y* direction (projection plane) and 24.5% in the *Z* direction (printing direction). Subsequently, the dimensions of the printed part were scaled up following the shrinkage ratio. To ensure printing accuracy, the layer thickness was set to 31.25 μm. Following the printing process, the parts underwent cleaning with a PPG solvent, using an ultrasonic cleaning machine to eliminate any residual material from the scaffolds. Subsequently, the scaffolds were subjected to degreasing at 560°C under argon protection to eliminate all polymer components (including monomer HDDA, crosslinker PPTTA, and PPG 400) from the materials. The temperature curves for both the degreasing and sintering processes are illustrated in Figures 2(a) and 2(b). A controlled temperature increase strategy was employed to reduce the risk of excessive pyrolysis of the resin, which could compromise the structural integrity of the parts and lead to cracks. In the degreasing process, the temperature was increased at a rate of 0.075°C/min from 400°C to 460°C and then at a rate of 0.25°C/min from 460°C to 560°C. Finally, a sintering process was implemented to achieve densification of the scaffold. The temperature was elevated at 4°C/min during the sintering process from room temperature to 1200°C. Subsequently, it was further increased to 1600°C at a rate of 3°C/min and maintained at that temperature for 2 h. Following this, the temperature was gradually reduced to 1200°C at a rate of 3°C/min and then brought down to room temperature at 4°C/min.

### 2.4. Characterization of Gyroid Scaffolds

The quality of scaffold fabrication was assessed using micro-CT (μCT) imaging, which offers a nondestructive approach to material characterization. The fabricated scaffold's porosity analysis was conducted using the Archimedean principle [37] and μCT scanning methods. For the Archimedean principle, the porosity is measured as follows: firstly, the sample is weighed in air and recorded as *M*_0_. Subsequently, the sample is placed in water to ensure complete immersion. The sample and water are heated in a drying oven until the water boils for 2 h, allowing the water to fully penetrate into the pores of the sample and removing air from the pores. After cooling to room temperature, the sample is hanged in a small basket and still submerged in water. The system is weighed again and recorded as *M*_1_. After that, the sample is taken out of water. After removing water from the surface of the sample, the weight is measured as *M*_2_. The porosity of the sample *P*_*o*_ is calculated using equation (2):

(2) $$\begin{array}{c} P_{o}=\frac{M_{2}-M_{0}}{M_{2}-M_{1}}. \end{array}$$

Both the Archimedean principle and μCT method are implemented to cross-validate the porosity measurements of the gyroid scaffold. The in vitro μCT system (Always Imaging, Ningbo, Zhejiang) was utilized for scanning samples, employing 80 μA and 130-kV accelerated voltages with a pixel size set of 15.6-μm resolution. The overall porosity of the scaffold was calculated using an algorithm (VG Studio, MAX 3.5) after applying an appropriate threshold to eliminate micropores. Additionally, the phase purity and crystallinity of the alumina ceramic scaffold were analyzed using XRD. The data of XRD test are first subjected to background subtraction and smoothing, followed by finding diffraction peaks. The obtained diffraction peaks are compared with the standard PDF card to determine chemical composition.

The compressive strength and compressive modulus of gyroid scaffold were measured through a compressive test conducted on a 10 mm × 10 mm × 10 mm cubic structure with a gyroid pattern. The porosity of the tested cubic sample was adjusted to match the porosity of the printed gyroid scaffold. A Universal Testing Machine (PLD5, Xi'an Lichuang Co., China) was employed to conduct the compression test. The cross-head speed was set at 1 mm/min. The compressive strength *σ*_*c*_ and compressive modulus *M*_*c*_ of the samples are calculated using equations (3) and (4), respectively,

(3) $$\begin{array}{c} \sigma_{c}=\frac{F}{S_{eq}}, \end{array}$$

(4) $$\begin{array}{c} M_{c}=\frac{F/S_{eq}}{\Delta L/L}, \end{array}$$

where *F* represents the maximum compressive force before breakage, *S*_*eq*_ represents the equivalent cross-sectional area of the sample, and Δ*L* and *L* represent the total deformation and total length of the sample, respectively. The microstructure of the gyroid scaffold was examined using a field emission scanning electron microscope (FESEM, Quanta 250FEG, FEI Co., America) with a scanning voltage of 20 kV.

### 2.5. Study of Rate of Drug Release

As detailed previously, 1 μg of vancomycin was thoroughly mixed into 2.0 wt% of type I collagen before the material was processed into gyroid ceramic scaffolds to assess the drug release rate. The polymer was then immersed in 5 mL of PBS (pH 7.4) at 37°C, and 200 μL of samples were collected at different times (1, 3, 7, 14, and 28 days) to measure the drug concentration. After collection, the same volume of PBS was added to the system to keep the total volume constant. Vancomycin concentrations were measured using an ELISA kit (Shanghai Enzyme-linked Biotechnology Company, China) according to the manufacturer's instructions. Each test was repeated five times for each sample, and the average value was calculated.

### 2.6. In Vitro Experiments

#### 2.6.1. Extract Preparation

According to the ratio of materials and extraction medium recommended in the literature standard [38], the material of 5 × 5 × 2 mm^3^ was extracted in a 37°C incubator for 24 h according to the ratio of a 1-cm^2^ sample surface area and a 10-mL extraction medium. The extraction medium was DMEM-F12 culture medium containing 10% calf serum. After leaching, the leaching solution was 0.22 m including the microporous filter, 4°C being saved for later use. (By the above methods, we obtained the following four groups of extracts: blank control group, scaffold group (G), scaffold-loaded collagen group (G-Col), and scaffold-loaded collagen, BMP2, and ZA group (G-Col-B-Z).

#### 2.6.2. Culture of Bone Marrow Mesenchymal Stem Cells (BMSCs) and Human Umbilical Vein Endothelial Cells (HUVECs)

Both MC3T3-E1 cells and HUVECs were purchased from the Chinese Academy of Sciences Shanghai Biochemistry of the College of Life Science and cultured in α-MEM supplemented with 10% FBS, 100 U mL^−1^ of penicillin, and 100 μg mL^−1^ of streptomycin. The medium was refreshed every 2 days. When cellular proliferation reached 80% confluence, cell passaging was performed. All cells were incubated at 37°C in a humidified incubator with 5% CO_2_.

#### 2.6.3. Study of Biocompatibility

The proliferation of BMSCs (Chinese Academy of Sciences Shanghai Biochemistry of the College of Life Science) was assessed using the Cell Counting Kit-8 (CCK-8) assay at 0, 1, 3, 5, and 7 days. The CCK-8 kit was purchased from Solarbio (Beijing, China). The control group consisted of BMSCs maintained in Dulbecco's modified Eagle's medium (DMEM) culture medium, which include sugars, amino acids, vitamins, and inorganic salts, while the experimental group was cultured with the material extract. The culture medium of fix samples (6 wells of a 96-well plate) was removed at each timepoint; cells were washed with PBS buffer solution, and 100 μL of serum-free DMEM medium containing 10% CCK8 was placed in direct contact with cells. Following incubation at 37°C for 3 h, the OD value of the supernatants was measured at a wavelength of 450 nm using an enzyme labeling instrument (spectrophotometer). The serum-free DMEM medium containing 10% CCK8 was used as the blank control.

#### 2.6.4. Cell Material Interaction Studies

Material–cell interactions were evaluated by performing scanning electron microscopy (SEM). Before cell seeding, the alumina films were sterilized by placing in 70% ethanol for 4 h, followed by washing once with phosphate-buffered saline (PBS) (1×). The films were incubated in complete medium (10% FBS and 1% penicillin–streptomycin antibiotic) for 1 h prior to seeding. For seeding, the cells were trypsinized and checked for viability (trypan blue exclusion assay), and a total of 1 × 10^4^ cells dispersed in 10 μL of complete medium were seeded on the film surface. The cell-seeded films were incubated at 37°Cin 95% RH and 5% CO_2_ environment, allowing cells to adhere on the film surface for 3−4 h followed by the addition of 500 μL of complete media in each well in a 24-well nontreated tissue culture multiwell plate. Cell adhesion on surfaces was estimated by performing SEM. Briefly, at day-3 post seeding, the cell-seeded films were washed with PBS (1×) and fixed using paraformaldehyde (4%) solution for 1 h at 4°C. The fixed cells were dried in a desiccator, gold−palladium sputter-coated (108 auto, Cressington), and imaged using SEM (EVO18, Zeiss) to evaluate cell morphology.

#### 2.6.5. Osteogenic and Angiogenic Gene Expression

To analyze the expression of osteogenic and angiogenic genes, quantitative real-time polymerase chain reaction (qRT-PCR) was conducted. Briefly, BMSCs/HUVECs were seeded into a 6-well plate at 1 × 105 per well and cultured in DMEM for attachment. After 24 h, the culture medium was replaced with the extracts supplemented with 0.25 mM ascorbic acid, 10 nM β-glycerophosphate, and 20 nM dexamethasone. The extracts were changed every 3 days. The PCR analysis was performed at day 7 for BMSCs and day 3 for HUVECs. At these time points, total RNA was extracted using the RNeasy mini kit (Qiagen, Duesseldorf, Germany), and complementary DNA (cDNA) was reverse-transcribed using All-in-One cDNA Synthesis Supermix (Bimake, Houston, TX, USA). qRT-PCR was performed using the 7500 Real-time PCR system (Applied Biosystems; Thermo Fisher Scientific, Waltham, MA, USA). The empty group was used as the control group, and GAPDH served as the house-keeping gene. The reaction conditions were 95°C for 35 s, followed by 40 cycles of 95°C for 15 s and 60°C for 45 s. Genetic expression was calculated using the 2^−△△CT^ formula.

#### 2.6.6. Wounding Healing Assay

The migration of HUVECs under the effect of scaffold extract was evaluated by scratch wound healing assay and transwell migration assay. The extracts of scaffolds were prepared according to previous studies [39]. HUVECs were seeded into 6-well plates at a density of 2 × 10^5^ cells/well and divided into four groups with two wells in each group. At the time of cell growth to approximately 90% confluence, a 100-μL micropipette tip was used to draw a straight line through the middle of each well. Each well was washed three times with PBS to remove cells in the scratch, and the pre-prepared extract medium was added to each well of each group, and DMEM medium containing the same amount of serum-free was added to the control group. At 0, 6, 12, and 24 h, scratch images were taken at 40× magnification in three fields, and images were saved. ImageJ software was used to measure the residual area to assess migration.

### 2.7. Animal Bone Defect Models of Critical Size and *In Vivo* Implantation Studies

This study assessed the impact of scaffold functionalization with bioactive agents on bone formation and defect healing. The evaluation involved implantation of non-functionalized scaffolds and functionalized scaffolds with bioactive factors for critical bone defects in the rabbit cranial (8 mm in diameter, height of skull full thickness about 2–2.2 mm), respectively. In addition, the TPMS structured scaffold further expanded the surface area and prolonged the release time of bioactive agents by perfusion with type I collagen. Twelve female Chinese white rabbits, aged between 2 and 3 months (approximately 2.5 kg weight), were distributed into four groups for critical size defect models, with each rabbit having four defects (see Table 2). The abbreviations utilized persist throughout the remainder of this article.

#### 2.7.1. Critical Size Defect in Rabbit Cranium

3D-printed gyroid scaffolds were sterilized with high-temperature steam (the temperature was 121.3°C, and the pressure was 103.4 kPa for 30 min). Collagen was introduced into the scaffold through a dual syringe extrusion under aseptic conditions. The scaffold is adaptable for direct use or can be filled with type I collagen combined with BMP-2 (5 μg/scaffold) and ZA (10 μg/scaffold). Local delivery of bioactive substances has been found to be effective in promoting bone formation and implant integration at doses lower than the systemic doses administered clinically. This low-dose delivery also eliminates the problem of dose-dependent side effects [40, 41]. BMP-2 were bought from Wuhan Fine Biotech Company (Wuhan, China). ZA was purchased from Novartis (Basel, Switzerland). The preparation of collagen and the specific methods of collagen-infused scaffolds are described in the Supporting Information, as depicted in Figures S1 and S2. Table 2 provides an overview of the condition of all study groups. Rabbits were anesthetized with 3% dose of pentobarbital (3.0 mL/kg) by ear margin intravenous injection. All animals received the prophylactic antibiotic ceftriaxone (40.0 mg/kg) and the analgesic tramadol (5.0 mg/kg) to ensure safety. Following hair removal from the head, the surgical area, approximately 5 cm in diameter, was disinfected with povidone-iodine. A full thickness skin incision was made from the frontonasal suture to the lambdoid suture, exposing the frontal and parietal bones. Upper tissue and periosteum were removed through scraping, exposing the underlying bone. A retractor was employed to restrict the skin and soft tissue, while a trephine, under continuous saline irrigation, was gradually used on both sides of the midline. This process created four full-layer circular defects, each approximately 8.0 mm in size. Two defects were made on the frontal bone and two on the parietal bone. Once the defect is created, the bone is carefully lifted and extracted. Any residual bone mass within the cavity is eliminated to prevent potential harm or irritation to the lower dura mater and the brain. The scaffolds are gently inserted into the cavity, ensuring no undue pressure on the underlying brain tissue. Following the designated groups outlined in Table 2, the scaffolds were implanted gently in the experimental group. The bioactive factors contained in the scaffold are naturally released with the loss and degradation of type I collagen in the body and directly contact the surrounding bone tissue. Subsequently, the skin was sutured using 3-0 silk thread (John-son & Johnson Medical (China) Ltd.), while the underlying periosteal tissue was left unsutured. Post-surgery, the animals received intramuscular injections of penicillin (5.0 mg/kg). The animals had unrestricted access to food and water throughout the recovery period.

#### 2.7.2. Analysis of Defects and Tissue Mineralization Through Radiological and Micro-CT Techniques

Following 120 days of implantation, the experimental animals were euthanized by excessive injection of pentobarbital, and samples of the cranium were collected. The cranium samples containing the implanted scaffolds were promptly preserved in neutral buffered formalin (4% w/v) (pH 7.4) for 48 h at 4°C. After fixation, the samples underwent a wash with deionized water and incubated in 70% v/v ethanol at 4°C for subsequent analysis. Digital x-ray images capturing tissue mineralization at the defect site were acquired through a μCT scanner. Rabbit cranium samples were subjected to μCT analysis (Always Imaging, Ningbo, Zhejiang). The imaging process utilized an energy setting of 130 kV and 80 μA, with 500 ms of x-ray irradiation, to capture CT micrographs of the rabbit cranium samples. 1440 projections were recorded, and the voxel size was 15.6 μm. In the reconstruction process, the scanned images underwent filtering with a Gaussian blur (2 pt radius). All images were rear-ranged and oriented to a uniform plane (VG Studio MAX 3.5) for analysis. Subsequently, quantitative morphological analysis was conducted on all samples (VG Studio MAX 3.5), applying a consistent grayscale threshold of 110 and 125 to the rabbit cranium samples. Circular regions of interest, each with a diameter of 8.0 mm and a height of the full thickness of the skull, were scrutinized on the rabbit cranium samples. To evaluate bone mass and microstructure, the bone volume ratio (BV/TV), trabecular separation/spacing (Tb.Sp), trabecular number (Tb.N), and trabecular thickness (Tb.Th) were calculated and analyzed.

#### 2.7.3. Analysis of Bone Formation and Defect Healing Through Histological Examination

We divided each specimen transversally into frontal and parietal parts, cutting longitudinally along the long axis of each specimen. So that each slice can get complete two defect coronal. The preserved samples underwent a gradual dehydration process, followed by resin embedding and generating hard tissue sections using a microscopical microtome (Leica SP 1600, Wetzlar, Germany) for subsequent histological analysis. The cranium sample, after embedding, was divided into two parts. Each part was halved transversely from the center of the defect, revealing coronal planes of two experimental areas on each slice. The initial slice width was approximately 300 μm, and after grinding, the final slice thickness was reduced to 50 μm. These sections underwent staining with hematoxylin and eosin (H&E) as well as Masson's trichrome (MT). The stained samples were analyzed to assess tissue infiltration, bone formation patterns, and scaffold integration.

#### 2.7.4. Statistical Analysis

In vitro experiments were carried out in triplicate, ensuring a minimum sample size of *n* = 3. For in vivo critical size defect studies, groups of three animals were involved, with four defects in each animal, maintaining a minimum sample size of *n* = 12. The experimental group, focusing on evaluating the effect of scaffold structure alone, had a sample size of *n* = 12, featuring four defects in each of the three animals. However, in the blank control group, one animal could only complete three defects due to anatomical limitations, resulting in a sample size of *n* = 3. For analysis of the micro-CT results, a paired two-tailed *t*-test or Wilcoxon test for paired samples was performed. Drug release and CCK-8's results were analyzed using an independent two-tailed *t*-test. All results are presented as means ± SD. ^∗^*p* < 0.05, ^∗∗^*p* < 0.01, and ^∗∗∗^*p* < 0.001 were considered significant.

## 3. Results

### 3.1. Characterization of Fabricated Scaffolds

**Figure 3:**
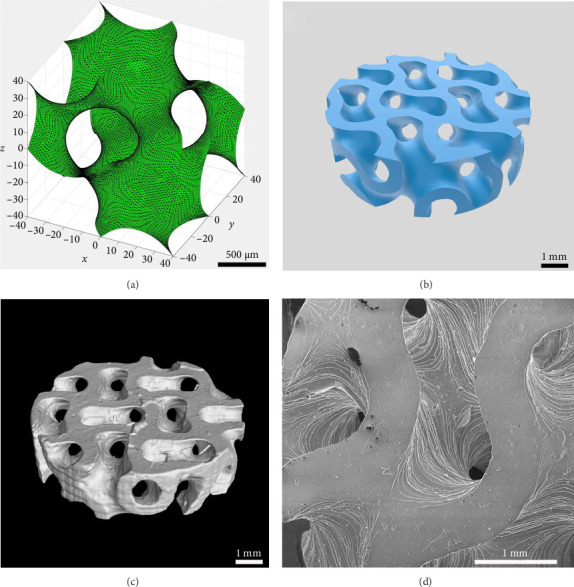
Images of the fabricated scaffold 3D models. (a) Gyroid unit. (b) Structural design of the scaffold. (c) Images of the fabricated scaffold 3D models rendered based on μCT. (d) SEM images of the gyroid scaffolds.

The average particle size of alumina powder was 150 nm, and the addition of Hypermer KD1 as the dispersant could prevent the sedimentation of the slurry and promote the dispersion of ceramic powder in the slurry [42, 43]. This yields the slurry with good stability. Before printing each layer, the scraper inside the printer will stir and flatten the slurry of the current layer [44], which can also promote the uniformity and stability of the ceramic slurry. The gyroid unit employed in this study was generated based on the iso-surface defined by (equation (1)), yielding a theoretical porosity of 60%.  Figure 3(a) displays the gyroid unit. The designed gyroid model for printing is depicted in Figure 3(b), while Figure 3(c) presents a 3D reconstruction of the gyroid scaffold. Using the Archimedes method on six printed scaffolds, the average porosity of the printed parts was determined to be 54.14%, with a standard deviation of 2.54%. This highlights the commendable consistency achieved through the DLP technology. Based on the Micro-CT reconstruction, the ceramic scaffold's total porosity is 54.9%. The average pore diameter of the gyroid scaffolds produced is 376 μm. The high-resolution SEM micrograph in Figure 3(d) illustrates the surface roughness of the fabricated scaffold. The results suggest a favorable outcome, indicating good consistency and desirable properties for the gyroid scaffolds.

#### 3.1.1. Mechanical Properties of the Scaffolds

**Figure 4:**
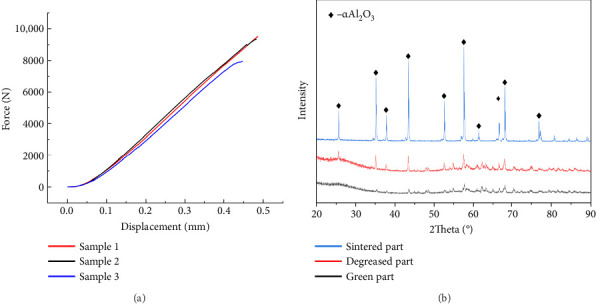
(a) Force–displacement curve of the sintered gyroid scaffolds. (b) XRD spectra of the individual ceramic scaffolds after 3D printing, degreasing, and sintering.

The compressive test results are illustrated in Figure 4(a). The maximum value of each curve is the maximum compressive force that each sample bears at breakage. The chosen ceramic material is alumina, known for its higher strength than the commonly used tricalcium phosphate material in tissue engineering scaffolds [45]. The gyroid scaffold exhibits an average compressive strength of 140.15 MPa and an average compressive modulus of 213.95 MPa. These findings emphasize the robust mechanical properties of the alumina-based gyroid scaffold. The porous structure of the scaffold contributes to a measured compressive strength for the 3D-printed alumina scaffold that is lower than that of a solid structure. Nevertheless, the obtained strength is considerably higher than that of the rabbit cranium, ensuring the scaffold's ability to endure within the rabbit cranium for an extended period without sustaining damage. This durability enables the observation of tissue regeneration around the scaffold over 120 days.

#### 3.1.2. Analysis of Scaffold Composition

The physicochemical composition of alumina ceramics was assessed using XRD. The results of these measurements are depicted in Figure 4(b). Following the standard PDF card, alumina material with good crystallinity displays x-ray diffraction characteristic peaks at approximately 25.58, 35.15, 37.78, 43.36, and 57.50 degrees [46]. The statistical analysis indicates that the characteristic peaks of the sintered parts align with those specified in the standard PDF card. This implies that the printing process and postprocessing do not compromise the purity of the material, and the sintered part maintains good crystal quality. In contrast, the green and degreased parts exhibit lower characteristic peaks and purity than the sintered parts. This outcome aligns with expectations, as un-sintered parts contain polymer components such as monomer, crosslinker, and solvent.

#### 3.1.3. Vancomycin Release Rate From Collagen-Filled Scaffold

**Figure 5:**
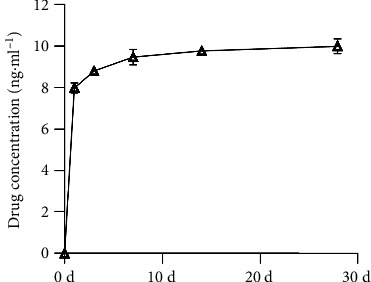
Rate of release of vancomycin loaded into collagen-filled scaffold.

As shown in Figure 5, the findings revealed that vancomycin displayed a burst release on the first day, increasing the concentration in the surrounding solution from 0 to 7.981 ± 0.243 ng·mL^−1^. Subsequently, the release rate stabilized, resulting in an observed concentration of 9.993 ± 0.356 ng·mL^−1^ observed after 28 days. This characteristic establishes a 2.0 wt% type I collagen as a valuable drug delivery system, facilitating the loading of therapeutic agents and ensuring continuous release.

### 3.2. In Vitro Studies Evaluated the Biological Properties of the Scaffolds

#### 3.2.1. Biocompatibility Studies and Cell Material Interaction Studies

**Figure 6:**
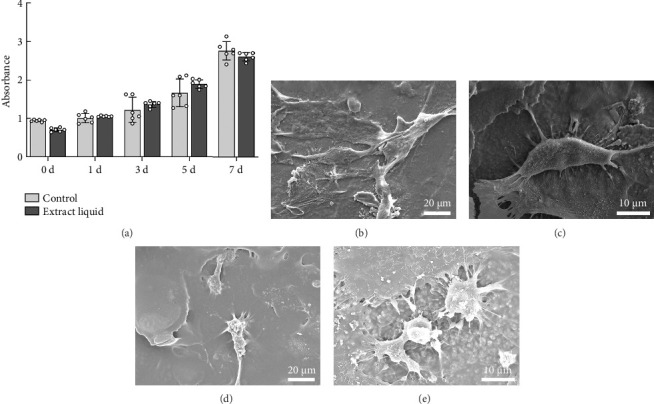
(a) CCK-8 kit for biocompatibility. (b–e) SEM imaging of human MSCs cultured on material films showing cell morphologies.

As depicted in Figure 6(a), the impact of material extracts on the proliferation of BMSCs was assessed using the CCK8 assay. The results revealed that, in comparison with the control group, the material extract exhibited no significant effect on the proliferation of BMSCs. This suggests that the alumina material employed in this study for scaffold construction demonstrates good biocompatibility. Through SEM, it was observed that alumina ceramic supporting cell adhesion, as observed by cells growing with highly extended and well-spread morphologies on material surfaces at day-3 post seeding (Figures 6(b), 6(c), 6(d), and 6(e)).

#### 3.2.2. The Scaffolds Enhance the Osteogenic Property of BMSC in Vitro

**Figure 7:**
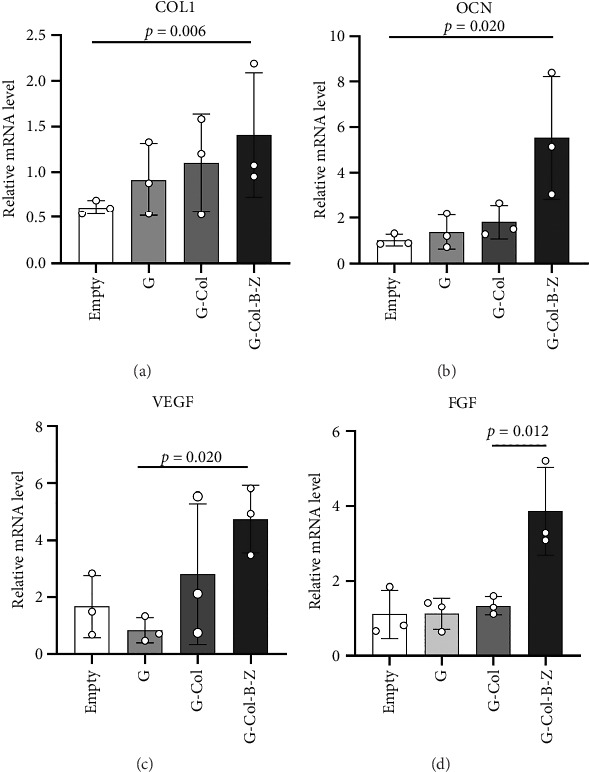
Osteogenesis of BMSCs and proangiogenic expressions of HUVECs cultured in extracts in vitro. This figure showed a fold increase of COL1 (a), OCN (b), VEGF, and (c) and FGF2 (d) normalized to housekeeping gene mus musculus glyceraldehyde-3-phosphate dehydrogenase (mGAPDH).

To assess the osteogenic properties of the scaffold in vitro, we conducted genetic level (PCR assays). In the PCR analysis, collagen type I (COL1), which regulates mineralization, increased by 2.6-fold in G-Col-B-Z at day 7 (Figure 7(a)). Osteocalcin (OCN), which regulates osteoblastogenesis, increased by 5.5-fold in G-Col-B-Z at day 7, significantly higher than that of G and G-Col (Figure 7(b)). The results showed that the expression of osteogenic genes COLI and OCN in the collagen-loaded group was significantly higher than that in the control group. However, the addition of BMP2 and ZA could further promote the expression, and there was a significant difference between the two compared with the control group.

#### 3.2.3. The Scaffolds Stimulate Proangiogenic Expressions of HUVECs in Vitro

According to the results, expressions of angiogenic genes were all upregulated in G-Col and G-Col-B-Z compared with the control group. For example, vascular endothelial growth factor (VEGF), which is responsible for vascular formation, was significantly upregulated, i.e., 1.3-fold in G-Col and 2.4-fold in G-Col-B-Z, and there was a statistical difference between the control group and G-Col-B-Z (*p* < 0.05). In addition, fibroblast growth factor 2 (FGF2), which accounts for cell proliferation, increased by 2.6-fold in G-Col-B-Z. Although there was no statistical difference between other groups. The results showed that collagen promoted the expression of angiogenic genes in endothelial cells after 3 days of culture, which was further enhanced by loading BMP2 and ZA. The difference was statistically significant (Figures 7(c) and 7(d)).

#### 3.2.4. Migration Ability of HUVECs Cultured in Vitro

**Figure 8:**
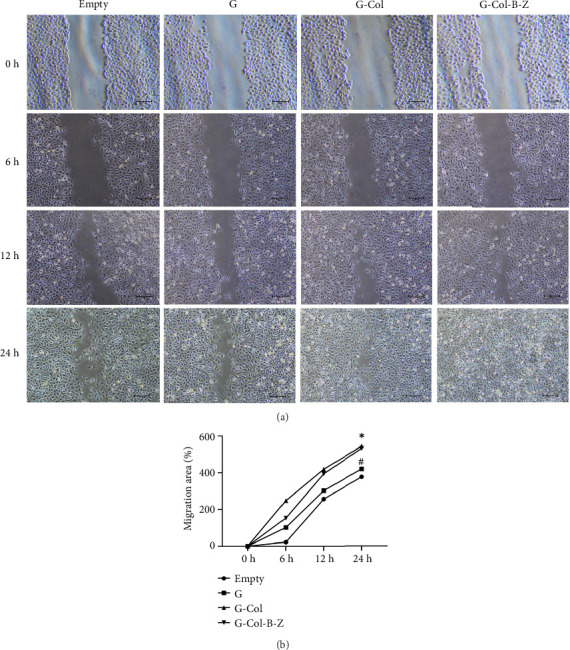
Effect of extracts on cell migration. (a) Wound healing assay of HUVECs. (b) Quantitative analysis of wound closure.

To further evaluate the proangiogenic effect of the scaffold, we performed a wound scratch healing assay to assess cell migration capacity (Figure 8). After incubation of the extract with HUVECs for 24 h, the scratch gap was 104 μm in the blank control group and 108.6 μm in the G group, and there was still a significant gap. The scratch gap of the G-Col group was 58.8 μm, and the scratch of these three groups did not heal completely. In contrast, the cells in the G-Col-B-Z group not only migrated faster but also healed completely at 24 h. The migration speed of endothelial cells in the G-Col-B-Z and G-Col groups was significantly higher than that in the blank control group (*p* < 0.05). After 24 h of incubation, HUVECs in the G-Col-B-Z group had completely covered the original scratch area, and the difference was statistically significant.

### 3.3. *In Vivo* Implantation and Mineralization Studies in Bone Defect Models

This study built a rabbit flat bone (cranium) defect model, and four control groups were built to investigate the impact of neat and functional composite scaffolds on bone formation. The study group was specifically designed to analyze the effects of scaffold implantation and empty defects, as well as the influence of BMP and ZA scaffold functionalization on the overall bone formation. They were divided into four groups, which were blank control group, pure scaffold group, scaffold group loaded with type I collagen, and functional scaffold group with BMP-2 and ZA (Table 2). Bone formation was evaluated 120 days after the implantation in the cranium model.

#### 3.3.1. Radiological and μCT Analysis of Tissue Mineralization

**Figure 9:**
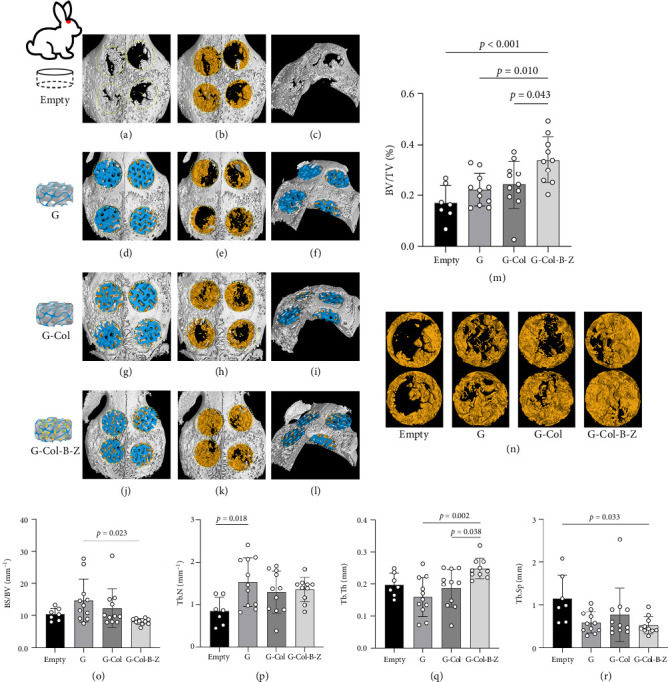
Bone formation analysis of cranial implants. (a–l) μCT 3D-rendered images representing defect margins (yellow circles) and implanted scaffolds in four groups. The scaffolds are visualized in blue or have been removed by thresholding to show tissue mineralization. (m, o–r) BV/TV, BS/BV, Tb.N, Tb.Th, and Tb.Sp within the defects in all groups presenting mean ± standard deviation. (n) Representative defects from each group showing mineralized tissue depositions and defect healing 120 days postimplantation.

The evaluation of bone formation involved both radiographic gross imaging and quantitative μCT analysis of the collected bone samples (Figure 9). The μCT analysis of cranial samples revealed incomplete healing of the defect in the untreated blank control group. Bone infiltration was observed around the defect, but a significant void remained inside the defect area. Conversely, more mineralized tissue infiltrates were evident in the scaffold-implanted study group, extending into the defect area. Mineralization occurred extensively throughout the scaffold, effectively filling the internal void. Upon removal of the scaffold for μCT analysis, the pattern of mineralized tissue deposition became more apparent (Figure 9). Our observations indicate that the G4 group exhibited more substantial bone formation than the other three groups (G-Col-B-Z group, BV/TV = 0.340 [0.202–0.493], *p* < 0.05). However, the other two scaffold groups showed a similar trend but did not reach statistical significance compared to the empty group (*p* > 0.05). The functionalized composite scaffolds loaded with BMP and ZA demonstrated the minimum trabecular separation (Tb. Sp = 0.520[0.276–0.958]) and exhibited a significant difference compared to the blank control group (*p* < 0.05). Notably, the results of the BS/BV ratio for the drug-loaded scaffold group were lower than those of the blank control group. This suggests that the drug-loaded scaffolds may have a more compact bone structure with a lower surface-to-volume ratio, potentially indicating less bone resorption or a more stable bone remodeling environment. Furthermore, in comparison to the pure scaffold group, the G-Col-B-Z group displayed higher bone trabecular thickness (*p* < 0.05). However, no statistically significant differences were observed between groups in the number of bone trabeculae (*p* > 0.05).

#### 3.3.2. Histological Analysis of Bone Formation and Defect

**Figure 10:**
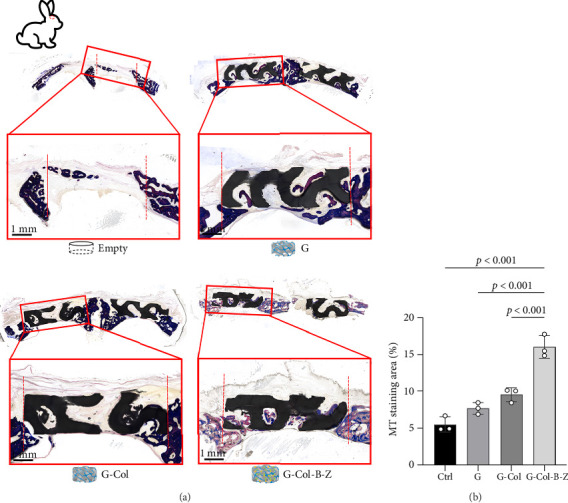
(a) MT staining analysis of bone tissue formation in cranium defects. The samples were transversely cut from the center of the defect, therefore showing two scaffolds in the images, one on the left and one on the right of the midline. Additionally, a magnification of one defect is shown. Red dotted lines represent original defect margins. (b) Quantitative results of tissue sections analyzed by ImageJ.

In the rabbit cranium study, composite scaffolds performed better than pure scaffolds (G) and blank controls (Figure 10). Both continuous and nodular mineralized tissue infiltration (depicted in dark blue) were more pronounced in the composite scaffolds, and bone infiltration throughout the defect appeared more uniform. In contrast to the blank control group, the scaffold group displayed a distinct pattern of tissue infiltration within the defect. The bone deposition in the scaffold group was higher and extended further into the scaffold structure. The scaffold group with type I collagen infiltration (G-Col) demonstrated less mineralized tissue infiltration. In contrast, the BMP + ZA-loaded scaffold group exhibited higher and more uniform mineralization. Among all experimental groups, the content of the BMP + ZA-functionalized composite scaffold (G-Col-B-Z) was the highest. Furthermore, compared with the blank and pure groups, more noticeable internal bone growth was observed in the scaffold group, characterized by small nodules deposited on the scaffold and thicker bone infiltrated from the periphery. However, a relatively complete new bone tissue infiltration appeared on the lower surface of all collagen-supported scaffolds.

**Figure 11:**
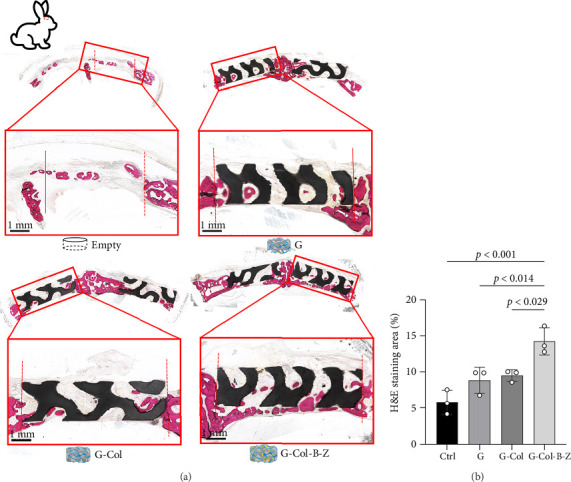
(a) H&E staining analysis of tissue infiltration and bone formation into the rabbit cranium defects. Red dotted lines represent original defect margins. (b) Quantitative results of tissue sections analyzed by ImageJ.

Moreover, the H&E staining results depicted hard tissue and soft tissue cell types and patterns infiltrating the defect area (Figure 11). The H&E analysis of rabbit cranium sections (Figure 11) revealed that in the blank control group, there was a small amount of bone infiltration around the defect margin, and it was relatively concentrated.

Defects containing scaffolds were typically filled with more mineralized tissue than the control group, and even the neat scaffold group (G) displayed significantly higher bone infiltration on scaffold surfaces and in pores. The degree of bone mineralization was slightly lower in the type I collagen-filled scaffold group without adding drugs and bioactive factors. However, incorporating BMP and ZA significantly increased the infiltration of hard tissue, resulting in thicker and more uniform walls throughout the scaffold, and stratified mineralization was observed. Compared with the other groups, the functional scaffold group exhibited the least soft tissue infiltration, and mineralized tissue infiltration significantly increased.

## 4. Discussion

In this study, we fabricated porous alumina ceramic composite scaffolds for bone regeneration using photocurable resin and DLP. Alumina has been recognized as a suitable material for bone regeneration owing to its biocompatibility and enough mechanical properties for bone implants [47]. It has already found applications in joint replacements and dental implants [48]. Indeed, being non-biodegradable, alumina allows for creating permanent scaffolds or implants. This feature enables a careful examination of the scaffold structure. The compressive stress of the gyroid scaffold is 140.15 MPa, which is equivalent to and slightly higher than that of human cortical bone (100 MPa) [49].

One of the advantages of the DLP method is its superior printing accuracy. The SEM image and 3D reconfiguration of the scaffold (see Figures 3(c) and 3(d)) reveal good surface quality. This ensures the precision of the scaffold structure experiment. Although the designed scaffold porosity is 60%, the measured porosity of the sintered scaffold is 54.9%. This discrepancy is partially attributed to the challenge of completely removing all residual ceramic slurry from the scaffold after printing using the PPG solvent and ultrasound cleaning. The dimensional deviation has consequently influenced the porosity. When there is a need to produce a scaffold with a specific porosity, the desired outcome can be achieved by adjusting the porosity of the designed digital model accordingly.

While there is no unanimous agreement on the ideal pore size for bone scaffolds, studies have suggested that osteoblasts' favorable pore sizes should be larger than 100 μm. In contrast, smaller pore sizes are associated with the formation of fibrous tissue [50]. Cheng et al. reported that a magnesium scaffold with a pore size of 400 μm facilitated the formation of mature bone tissue by promoting angiogenesis [51]. Small pores in the range of 50–100 μm are known to induce endochondral ossification, while larger pores in the range of 100–300 μm are conducive to induce intramembranous ossification. Furthermore, these larger pores also facilitate waste clearance and nutrient supply [52]. The gyroid scaffold designed in this study possesses a pore size of approximately 376 μm, which was a favorable pore size for the transplantation in this study [53] and subsequently utilized in the later in vivo experiments. The composite scaffolds were further infiltrated with rat tail type I collagen to enhance the bone conduction surface area for cell infiltration while supporting the locally controlled release of various bioactive molecules. Type I collagen, a hydrophilic material, is frequently employed in tissue engineering research related to bone regeneration [54, 55].

In this study, one of the main goals was to augment the bone-inducing properties of the gyroid structure by incorporating BMP (5 μg/scaffold) and ZA (10 μg/scaffold) and to evaluate its potential for in vivo drug delivery. These dosages, known to be effective for local bone induction, are significantly lower than the clinically employed systemic doses [56].

BMP and ZA can be absorbed into type I collagen and onto the surface of ceramic materials, facilitating the local delivery of bioactive molecules. The synergistic cooperation between BMP and ZA involves BMP inducing increased bone deposition and ZA mediating the inhibition of osteoclast-mediated absorption, ultimately leading to an overall enhancement in bone deposition. We introduced a TPMS structure with high connectivity in this experiment. We hypothesize that ceramic scaffolds with the TPMS structure (type gyroid) would offer ample mechanical support, and their smooth surface structure and high connectivity would facilitate load distribution, bone entry, type I collagen infiltration, and functionalization with BMP and ZA, consequently enhancing mineralization [57]. Compared with the blank control group, G, G-COL, or G-COL-B-Z scaffolds were introduced into a rabbit cranial defect model of critical size ∼8 mm to assess cell infiltration and bone-supporting abilities. Micro-computed tomography (μCT) proved effective in healing, demonstrating notable defect bridging in both groups. Nonetheless, histological examination suggested that the G-Col-B-Z scaffold displayed a more substantial presence of thick-walled mineralized tissue penetrating from the surrounding region into the scaffold. Moreover, histological analysis revealed the presence of nodular fusiform mineralized tissue deposits across the surface of the scaffold, transforming into mature lamellar bone. Despite having similar bone conduction surfaces, this phenomenon was conspicuously absent in the G scaffold, indicating a lack of bone induction cues in the latter. Confirmation of this observation came from μCT analysis, where the G-Col-B-Z group displayed a higher average BV/TV of 0.340 [0.202–0.493] for quantitative mineralized tissue deposition. Additionally, smaller bone trabecular separation compared to other groups was noted, signifying superior bone formation in the G-Col-B-Z group.

In a 120-day experiment of a critical-size rabbit cranial defect model across four groups, the composite scaffold functionalized with bioactive molecules (BMP + ZA) exhibited greater mineralized tissue deposition than the non-functionalized scaffold. This highlights the gyroid scaffold's capacity to protect and support the defect while offering a substantial surface area for bone tissue growth. Examination of histological sections indicated that bone ingrowth was possible from the existing bone margin, with collagen-filled areas in the scaffold's lower surface guiding bone infiltration. This pattern of bone formation likely contributes to the observed higher overall bone formation as revealed by μCT. In the blank control group (control), fibrous tissue occupied the defect, which remained incomplete and unbridged. In contrast, although total mineralized tissue infiltration was similar between pure ceramic scaffolds (G) and blank controls (control), the infiltration patterns differed. In the G group, bone formation was more dispersed, indicating a more robust bone conduction surface. The infiltration patterns of bone were nearly the same in both the collagen-infiltrated (G-Col) group and the ceramic scaffold-only (G) group, showing a slightly higher final bone mass in the G-Col group, though lacking significant statistical difference.

While the volume of bone formation in gyroid ceramic scaffold with type I collagen was slightly higher than that in the pure ceramic scaffold (G) and blank control groups, the MA- and HE-stained sections disclosed that bone formation was concentrated at the periphery and less within the scaffold. We interpret this as the filling of type I collagen diminishing the overall porosity of the scaffold. Without a robust induction signal, collagen filling may be seen as spatial occupation, impeding cell infiltration and reducing new bone deposition in the collagen-infiltrated scaffold (G-COL). However, when the same collagen was enriched with the bioactive molecules BMP-2 and ZA, μCT and histological analyses revealed significantly enhanced bone deposition. This observation supports the idea that combining a bone conduction surface and potent bone-inducing factors (BMP + ZA) positively influences cell differentiation and mineralized tissue deposition. The TPMS ceramic scaffolds produced through DLP offer a framework for composite filling and bioactive factor loading. The gyroid structure's high porosity and extensive surface area provide ultra-high connectivity and loading capacity, making it an excellent bone conduction surface for bone growth. Sintered alumina ceramics exhibit extremely high hardness and toughness, making them ideal for supporting cranium healing as they can better protect the underlying soft brain. The suitable hardness provided by ceramics also stimulates progenitor cells to differentiate into osteogenic lineages, promoting osteogenic induction. Furthermore, since the alumina-sintered ceramics utilized in this study do not degrade, it circumvents the ceramic powder generated by degradation, potentially causing inflammation [58]. However, this is not fully representative of the clinical application and similar experiments on bigger animals would be necessary in order to validate such a treatment concept in more realistic conditions.

## 5. Conclusion

Within the limits of the present study, we successfully prepared gyroid-structured alumina ceramic scaffolds with specific porosity by means of DLP 3D printing. The feature of the designed scaffold is successfully replicated. In addition, by incorporating BMP- and ZA-functionalized scaffolds, we improved the osteogenic induction ability of the scaffolds and could observe the osteogenic ability of the functionalized scaffolds by the osteogenic efficiency in vivo. The collagen-filled scaffold delivery of these molecules can decrease their required therapeutic doses and overcome inefficient delivery-related side effects. The study provides strong evidence in favor of 3D-printed composite with the ceramic TPMS structure and bioactive factors as synthetic bone substitutes.

## Figures and Tables

**Table 1:** Composition of the alumina ceramic slurry.

Components	Materials	Concentration
Alumina powder	Al_2_O_3_	40 vol% of ceramic slurry

Premix	HDDA	15 vol% of ceramic slurry
	PPTTA	21 vol% of ceramic slurry
	PPG 400	24 vol% of ceramic slurry

Dispersant	Hypermer KD1	2 wt% of alumina powder

Photoinitiator	TPO	1.5 wt% of premix

**Table 2:** Study groups within the in vivo critical size defect study.

Group	Treatment	No. of animal	No. of defects
Empty	Empty	3	11
G	Gyroid	3	12
G-Col	Gyroid + collagen type I	3	12
G-Col-B-Z	Gyroid + collagen type I + BMP-2+ZA	3	12

## Data Availability

The data that support the findings of this study are available on request from the corresponding authors. The data are not publicly available due to privacy or ethical restrictions.

## Supporting Information

We have added a Supporting Information to this paper, in which we describe in detail how to obtain the collagen material we used and how to integrate the collagen into the ceramic scaffold together with bioactive factors.
