# Supplementary material for: 3D-Printed Triply Periodic Minimal Surface Ceramic Scaffold Loaded With Bone Morphogenetic Protein-2 and Zoledronic for Cranium Defect Repairment
Source: J Tissue Eng Regen Med. 2025 May 26;2025:9964384. doi: 10.1155/term/9964384 (PMC12129610; doi:10.1155/term/9964384)
Supplement: Supporting Information — Additional supporting information can be found online in the Supporting Information section. [file 9964384.f1.docx]

**1. The preparation process of rat tail type I collagen**

The first type of glue used in this experiment was rat tail collagen. The specific preparation method is as follows: First, the tails of 5 SD rats frozen at -80 ° C were removed and thawed in 75% alcohol. Use tissue scissors to cut and tear the skin of the rat tail, use medical hemostatic forceps to break the bone of the peeled rat tail one by one along the thin end of the rat tail, and pull out the tendon inside the rat tail through the broken bone, repeat the above operations until all the tendons connected to the rat tail bone are pulled out. The tendons attached to the tail bone of each rat were cut and collected with scissors, placed in a large beaker, and the tendons were cut into pieces and cleaned successively with 75% alcohol and deionized water. Add 500 ml 0.1%(v/v) glacial acetic acid (A116116-500 ml, Aladdin Company) into the beaker and stir for 3 days using the mechanical mixer as shown in Figure 3-1A to fully dissolve the collagen in the rat tail tendon into the solution. The stirred viscous solution was divided into 50 ml centrifuge tubes, centrifuge was used (HC-3018R, Anhui Zhongke Zhongjia Scientific Instrument Co., LTD.), centrifuge was performed at a centrifuge temperature of 4℃, centrifuge speed of 4000 rpm, and time of 2 hours. The centrifugation results are shown in Figure 3-1b. Undissolved pieces of the rat tail tendon were found deposited at the bottom of the centrifuge tube. Carefully pour out the supernatant in the centrifuge tube, and discard the sediment in the centrifuge tube, as shown in Figure 3-1c. Finally, the supernatant is distributed in some thin open containers, such as iron lunch boxes, disposable tinfoil lunch boxes, etc., when packaging, the height of the supernatant in the container is best not to be too high, about 5 mm is the best. The container with the supernatant is pre-frozen in the refrigerator at -80℃, and after about 2 hours, the container with the supernatant is taken out, at which time the supernatant has condensed into a solid. The solid supernatant was put into a freeze-drying machine (SCIENTZ-12ND, Ningbo Xinzhi Biotechnology Co., LTD.) together with a container and freeze-dried for 72 hours to obtain the collagen foam, as shown in Figure 3-1d. The collagen foam can be stored in the refrigerator at -80℃ for a long time when not in use.


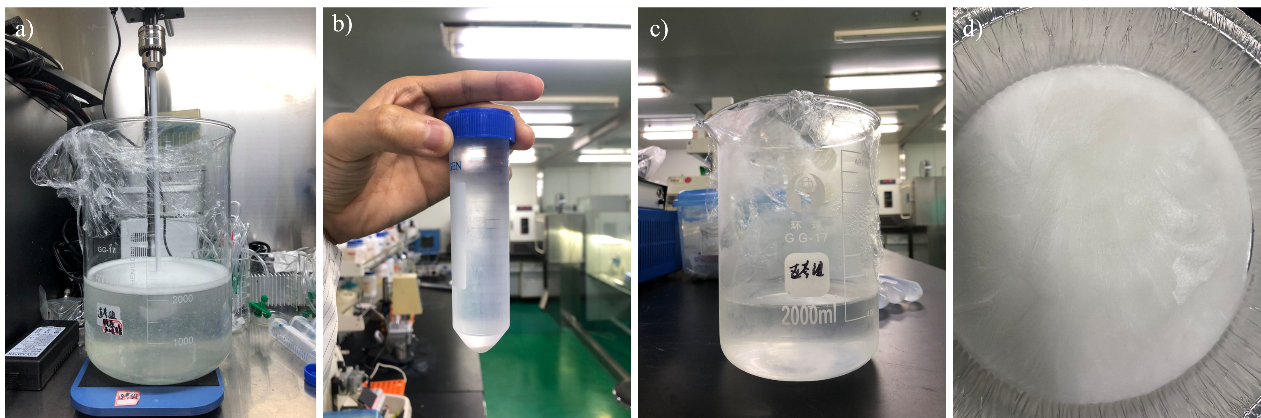


**Figure S1**. The preparation process of rat tail type I collagen: (a) mechanical stirring. (b) Centrifuge. (c) Supernatant. (d) Collagen foam.

**2. The preparation process of collagen perfusion into the scaffold.**

In this experiment, two docked syringes were used to simultaneously complete collagen dilution and perfusion through multiple extrusion, as shown in Figure S2. The syringes used were threaded syringes (5ml, Conde la), and a Ruhr external rotary straight butt joint was used to connect the two syringes. Due to the acidity of collagen, the color of the PH indicator in the culture solution changes from pink to yellow. In order to meet the conditions of collagen formation at PH neutral and 37 ° C, the PH in collagen was adjusted by 1M sodium hydroxide solution (306575-100G, Sigma-Aldrich). Unscrew the connecting part of the two syringes, add 5μl sodium hydroxide solution each time, connect the two syringes again, and squeeze several times until the ink color changes from yellow to light pink to prove that the PH is adjusted to neutral, as shown in Figure a. Take 2ml of collagen with a neutral pH, and the other syringe contains multiple scaffolds and 2ml of cell culture solution. The total amount of drugs and active factors can be calculated according to the required final concentration and added to the syringe where the scaffold is located. After completely draining the air from the two syringes, butt the two syringes with a Ruhr joint and squeeze them several times. When the culture solution was completely integrated with the collagen, it was naturally injected into the scaffold, as shown in Figure b. The collagen-wrapped scaffold in the syringe was extracted and placed in the petri dish. The neutral collagen would solidify rapidly at room temperature, as shown in Figure c. Wait for collagen to solidify and then cut out the scaffold with a surgical blade, as shown in Figure d.


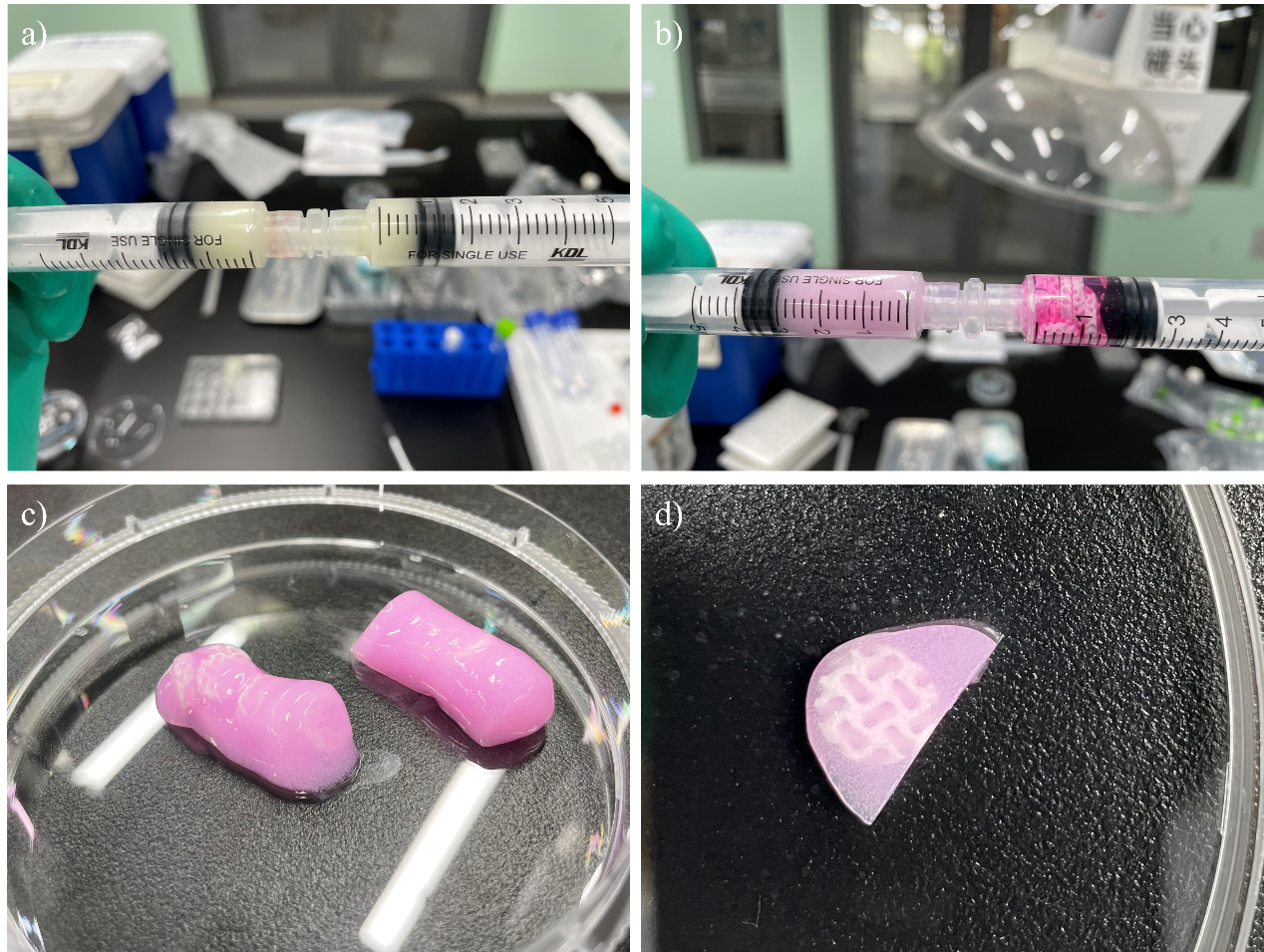


**Figure S2**. The preparation process of collagen perfusion into the scaffold: (a) Adjust collagen pH to neutral. (b) Collagen was injected by squeezing the syringe. (c) Remove the collagen-wrapped scaffolds from the syringe. (d) Cut out the post-perfusion scaffold.
